# Proteome-wide landscape of solubility limits in a bacterial cell

**DOI:** 10.1038/s41598-022-10427-1

**Published:** 2022-04-21

**Authors:** Ádám Györkei, Lejla Daruka, Dávid Balogh, Erika Őszi, Zoltán Magyar, Balázs Szappanos, Gergely Fekete, Mónika Fuxreiter, Péter Horváth, Csaba Pál, Bálint Kintses, Balázs Papp

**Affiliations:** 1 HCEMM-BRC Metabolic Systems Biology Lab, Szeged, Hungary; 2 Biological Research Centre, Institute of Biochemistry, Synthetic and Systems Biology Unit, Eötvös Loránd Research Network (ELKH), Szeged, Hungary; 3 Doctoral School in Biology, Faculty of Science and Informatics, University of Szeged, Szeged, Hungary; 4 Biological Research Centre, Institute of Plant Biology, Eötvös Loránd Research Network (ELKH), Szeged, Hungary; 5 Department of Biomedical Sciences, University of Padova, Padova, Italy; 6 Laboratory of Protein Dynamics, University of Debrecen, Debrecen, Hungary; 7 Institute for Molecular Medicine Finland-FIMM, Helsinki Institute of Life Science-HiLIFE, University of Helsinki, Helsinki, Finland; 8 HCEMM-BRC Translational Microbiology Research Group, Szeged, Hungary; 9 Department of Biochemistry and Molecular Biology, University of Szeged, Szeged, Hungary

**Keywords:** Proteins, Protein aggregation, High-throughput screening, Escherichia coli, Single-cell imaging

## Abstract

Proteins are prone to aggregate when expressed above their solubility limits. Aggregation may occur rapidly, potentially as early as proteins emerge from the ribosome, or slowly, following synthesis. However, in vivo data on aggregation rates are scarce. Here, we classified the *Escherichia coli* proteome into rapidly and slowly aggregating proteins using an in vivo image-based screen coupled with machine learning. We find that the majority (70%) of cytosolic proteins that become insoluble upon overexpression have relatively low rates of aggregation and are unlikely to aggregate co-translationally. Remarkably, such proteins exhibit higher folding rates compared to rapidly aggregating proteins, potentially implying that they aggregate after reaching their folded states. Furthermore, we find that a substantial fraction (~ 35%) of the proteome remain soluble at concentrations much higher than those found naturally, indicating a large margin of safety to tolerate gene expression changes. We show that high disorder content and low surface stickiness are major determinants of high solubility and are favored in abundant bacterial proteins. Overall, our study provides a global view of aggregation rates and hence solubility limits of proteins in a bacterial cell.

## Introduction

Maintaining solubility is a key requirement for proper functioning of proteins. It has been proposed that proteins are generally expressed close to their solubility limits under physiological conditions^1,2^. As a consequence, proteins are prone to misfold and aggregate when overexpressed due to the increased probability of forming intermolecular contacts that favor the aggregated state over the folded state^3^. For example, heterologous expression of recombinant proteins often leads to the formation of aggregates (i.e. inclusion bodies) in bacteria^4^.

Prior in vitro and computational studies revealed extensive variability of intrinsic aggregation rates between proteins^5–8^. Such differences likely influence whether aggregation occurs during protein synthesis (co-translationally^9^) or at a later stage (post-translationally) in the crowded intracellular environment. In particular, some proteins are prone to aggregate after reaching their folded states^10–14^. Aggregation from the folded state may occur through local unfolding events or structural fluctuations^11^ and appears to be prevalent among human amyloid proteins^15^. Therefore, understanding the rate and timing of aggregation have relevance to various fields from protein deposition diseases^11,12^ to the design of proteins with enhanced kinetic stability^16^. Despite its fundamental importance, however, we have only a limited understanding of which proteins are prone to aggregate early, potentially during synthesis, or post-translationally under in vivo conditions. Addressing this gap requires a proteome-wide study of aggregation rates in the intracellular environment.

**Figure 1:**
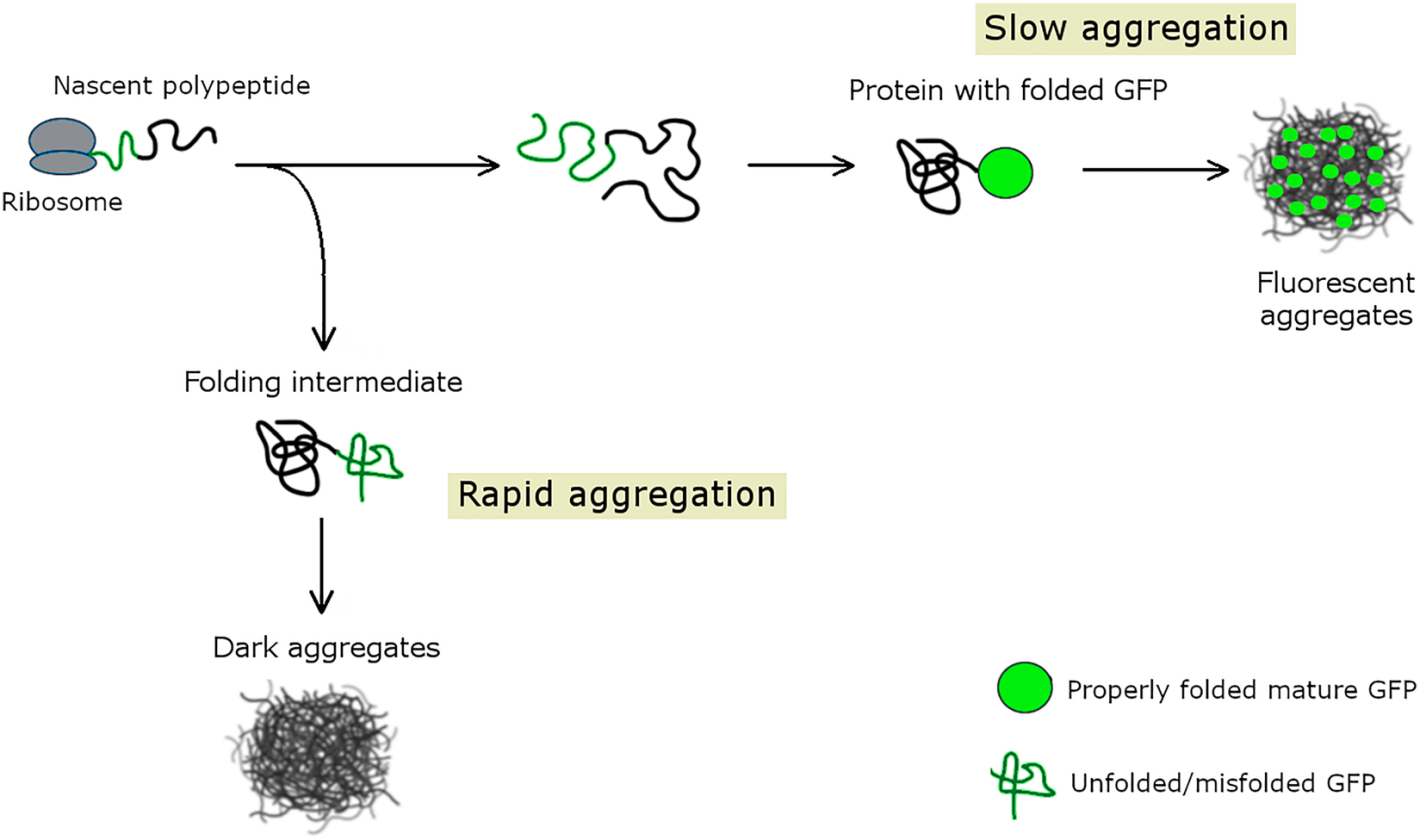
Distinguishing between rapidly and slowly aggregating proteins using GFP fusion. A C-terminally fused GFP tag reports on the relative time of aggregation across proteins. Nascent polypeptides with very high aggregation rates form anomalous intermolecular interactions before the GFP chromophore is committed to form^17^ and therefore the fused GFP tag would show no fluorescence, yielding dark aggregates (lower pathway, black ball). Note that this route of aggregation likely occurs before the protein is folded, possibly as early as translation. Proteins that aggregate after the GFP chromophore is committed to form would result in fluorescent aggregates (upper pathway) (de Groot & Ventura, 2006). These proteins aggregate relatively slowly, after being fully synthesized (i.e. post-translationally).

Here we describe a high-throughput in vivo approach that systematically classifies the cytosolic proteome into soluble, rapidly and slowly aggregating proteins in the model bacterium *Escherichia coli* (Fig. 1). In this system, we overexpressed all proteins at similar high levels thereby allowing comparison of the in vivo aggregation propensity across the whole proteome. The fate of overexpressed proteins was monitored by a GFP tag fused to the C-terminal of each protein. Such a GFP tag informs on the solubility status and relative time of aggregation of the upstream cytosolic protein^17–19^. Specifically, while a lack of fluorescent signal indicates misfolding and aggregation of the upstream protein before the GFP chromophore is committed to form, a strong fluorescent signal indicates properly folded, soluble fused upstream proteins^17^. Whereas GFP has long been used to distinguish between these two groups of proteins, case studies revealed a third phenotypic group of GFP-fused proteins that form fluorescent aggregates^19–21^. In particular, while rapid aggregation of the upstream peptide results in non-fluorescent aggregates, slow aggregation allows correct folding of the GFP moiety and yields fluorescent aggregates^19^. Therefore, the C-terminal GFP fusion informs on the relative time of aggregation, i.e. whether aggregation occurs early, potentially during synthesis, or post-translationally, after the downstream GFP tag has been fully synthesized and folded.

Based on these considerations, we used high-content microscopy to evaluate the intensity and subcellular distribution of fluorescence signals in order to distinguish between overexpressed proteins that (i) remain highly soluble, (ii) aggregate rapidly, possibly co-translationally (aggregates without GFP signal) or (iii) aggregate later, post-translationally (fluorescent aggregates). We then applied a data mining approach to systematically identify protein features that distinguish between these classes of proteins.

Our proteome-wide analysis reveals that most aggregation-prone cytosolic proteins form aggregates after translation. We find that such proteins exhibit low predicted aggregation rates from the unfolded state, tend to fold rapidly and interact more frequently with the chaperone DnaK as compared to those aggregating rapidly in vivo. Together, these features indicate a kinetic competition between aggregation and folding of the nascent polypeptide and raise the possibility that many proteins might aggregate after folding. Furthermore, we show that a substantial fraction (~ 35%) of the proteome remain soluble at concentrations much higher than those found naturally, indicating that many bacterial proteins are not expressed at levels close to their solubility limits. High disorder content and low surface stickiness are major determinants of such high solubility and ultimately influence the functioning of abundant proteins. Overall, our work provides a global view of aggregation rates and hence solubility limits of proteins in a bacterial cell.

## Results

### Proteome-wide classification of rapidly and slowly aggregating proteins in vivo

**Figure 2:**
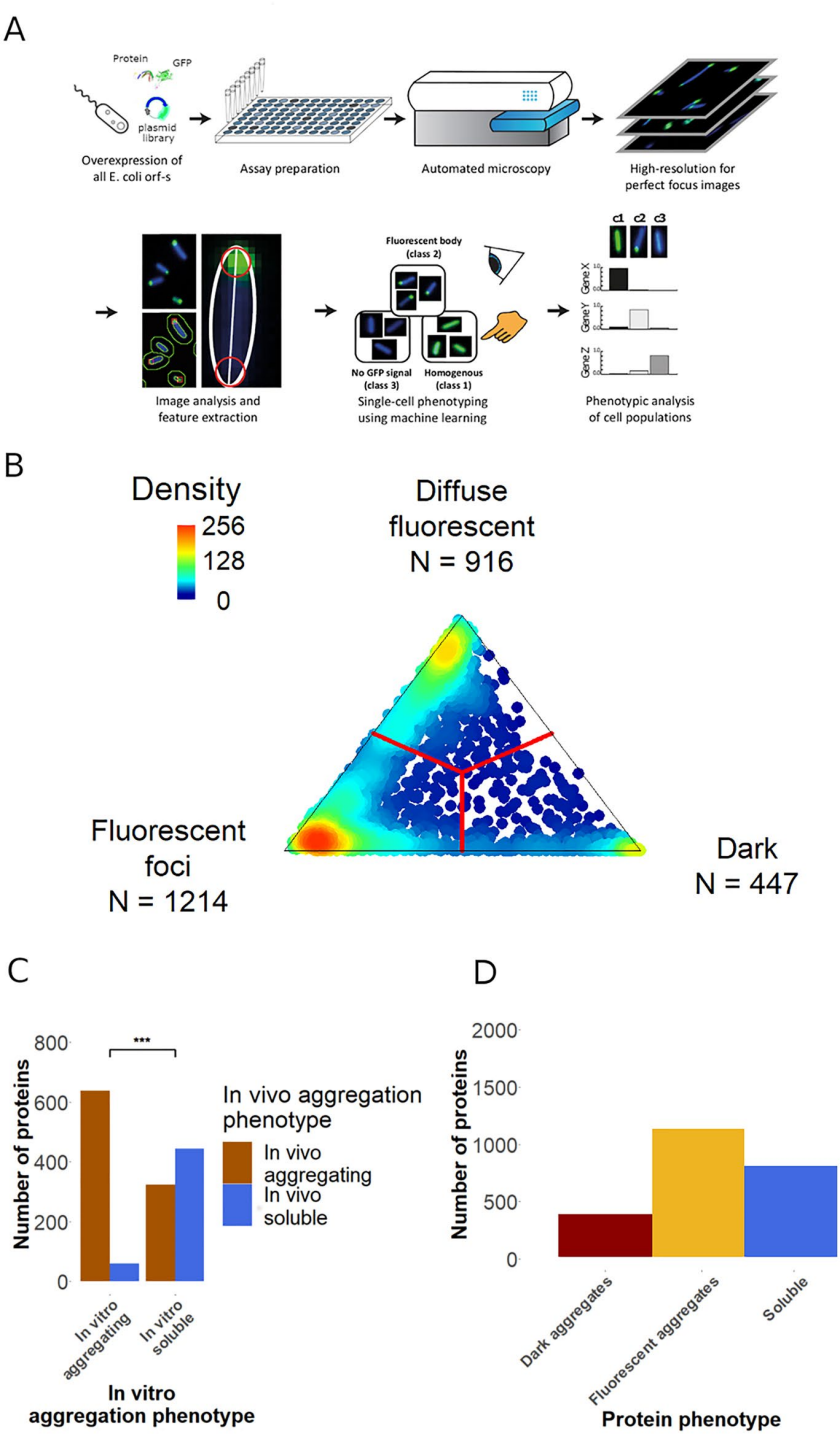
Experimental workflow and distribution of protein aggregation phenotypes. (**A**) Workflow of high-throughput protein solubility measurement and classification. (**B**) Distribution of cellular fluorescence phenotypes based on 1,000 classified cells for each overexpressed cytoplasmic protein (represented by dots). The location of each dot is calculated from the fraction of cells showing each phenotype, with red lines representing the decision boundaries between aggregation categories assigned. The majority of proteins are located close to the vertices, demonstrating that cells typically show homogenous aggregation behavior. (**C**) Comparison of in vivo and in vitro solubility phenotype of proteins. In vitro aggregating proteins show a strong overlap with proteins forming either dark or fluorescent aggregates in vivo (odds ratio = 14.5, P < 10^–10^, Fisher’s exact test). (**D**) Frequency of proteins according to their aggregation phenotypes.

We probed the solubility of 2577 cytosolic *E. coli* proteins upon overexpression at the single-cell level, using an image-based high-throughput screen (Fig. 2A). To this end, we monitored the fate of overexpressed proteins using a GFP fused to the C-terminal of each protein^22,23^. The intensity and subcellular distribution of the fluorescence signal is indicative of the aggregation rate of the upstream protein. Specifically, we distinguish between three classes of proteins. First, a homogeneous GFP signal dispersed uniformly in the cells indicates a folded and soluble protein^9^. Second, proteins without a GFP signal indicate rapid aggregation that occurs before the GFP chromophore is committed to form^9,17,19^. These aggregates, referred to as ‘dark aggregates’, therefore likely correspond to aggregation during or immediately after translation. Third, proteins with fluorescent foci represent aggregation that occurs after the GFP chromophore is committed to form^19^, indicating a slower rate of aggregate formation compared to proteins without a GFP signal. We refer to these aggregates as ‘fluorescent aggregates’.

After growing each overexpression strain in 96 well-plates, we applied a supervised machine-learning approach that automatically analyzes the images of approximately 1000 cells for each overexpression strain (Fig. 2A, Figure S1, Methods). Then, each overexpressed protein was classified into one of the above described three classes according to its predominant cellular phenotype: soluble, dark aggregate and fluorescent aggregate (Fig. 2A,B, Table S1). The frequency of cellular phenotypes and the resulting classification is shown in Fig. 2B.

To validate the accuracy of our high-throughput workflow, we took two complementary approaches. First, we assessed the overlap between the in vivo observed aggregation phenotypes and the in vitro measured solubility of the *E. coli* proteome, which was previously determined by expression using an in vitro reconstituted translation system^8^. Despite large differences in the in vivo and in vitro conditions, we found a strong overlap between the two datasets (odds ratio = 14.5, P < 10^–10^, Fig. 2C). In particular, 85.6% of the proteins that aggregate in vitro also show aggregation in our screen (Fig. 2C). Because the in vitro dataset was generated without a GFP tag, the strong agreement between the two datasets indicates that the C-terminal GFP tag does not significantly influence the solubility of the fusion partners. Furthermore, when proteins aggregating in vivo were grouped on the basis of their fluorescence phenotypes (i.e. dark and fluorescent aggregates), the overlap between in vitro and in vivo solubility remained equally high across the two categories (Figure S1). Thus, the GFP tag is unlikely to bias towards one or the other class of aggregate. Second, to rule out that the expression levels of the proteins in the dark aggregate class are below the fluorescence detection limit, we measured the expression of a representative set of cytosolic proteins by western blot analysis (see Methods; Table S2). We found that 92% of the tested proteins (i.e. 92 out of 100) from the dark aggregate class could be detected in their full-length forms and showed comparable protein levels with those classified as fluorescent aggregates (Figure S2A and B). This is consistent with a previous study demonstrating that GFP-fused overexpressed proteins are generally well-expressed in the cell, even in the absence of a fluorescent signal^9^. The detection of the vast majority of dark proteins also confirms their relative stability in the cell: although some degradation is possible for these proteins, a significant amount persisted during the course of the assay. To validate the solubility state inferred from the image-based classification, we next carried out western blot analysis after separating the soluble and insoluble fractions of 46 protein overexpressions, representing 18 dark aggregates, 14 fluorescent aggregates and 14 soluble proteins from our screen. Reassuringly, 100% and 88.9% of proteins classified as fluorescent and dark aggregates, respectively, are indeed predominantly present in their aggregated forms inside the cells (Figure S2C, Table S3). Furthermore, ~ 93% (i.e. 13 out of 14) of proteins classified as soluble by the image-based screen are indeed present in the soluble cellular fraction (Figure S2C). Taken together, these analyses indicate that our dataset is suitable to study the in vivo aggregation phenotype of overexpressed *E. coli* proteins on a proteomic scale.

Our screen reveals that only 18.6% of the cytoplasmic proteins form dark aggregates, while 37.8% and 43.6% of them remain soluble and form fluorescent aggregates, respectively (Fig. 2D). The presence of fluorescent aggregates in a strain overexpressing a particular protein implies that at least a subpopulation of protein molecules remains soluble while the C-terminally fused GFP is being synthesized and folded. Thus, our screen indicates that most (70%) aggregation-prone cytosolic proteins display relatively slow aggregation rates and are unlikely to aggregate co-translationally.

### Slowly aggregating proteins tend to fold rapidly

**Figure 3:**
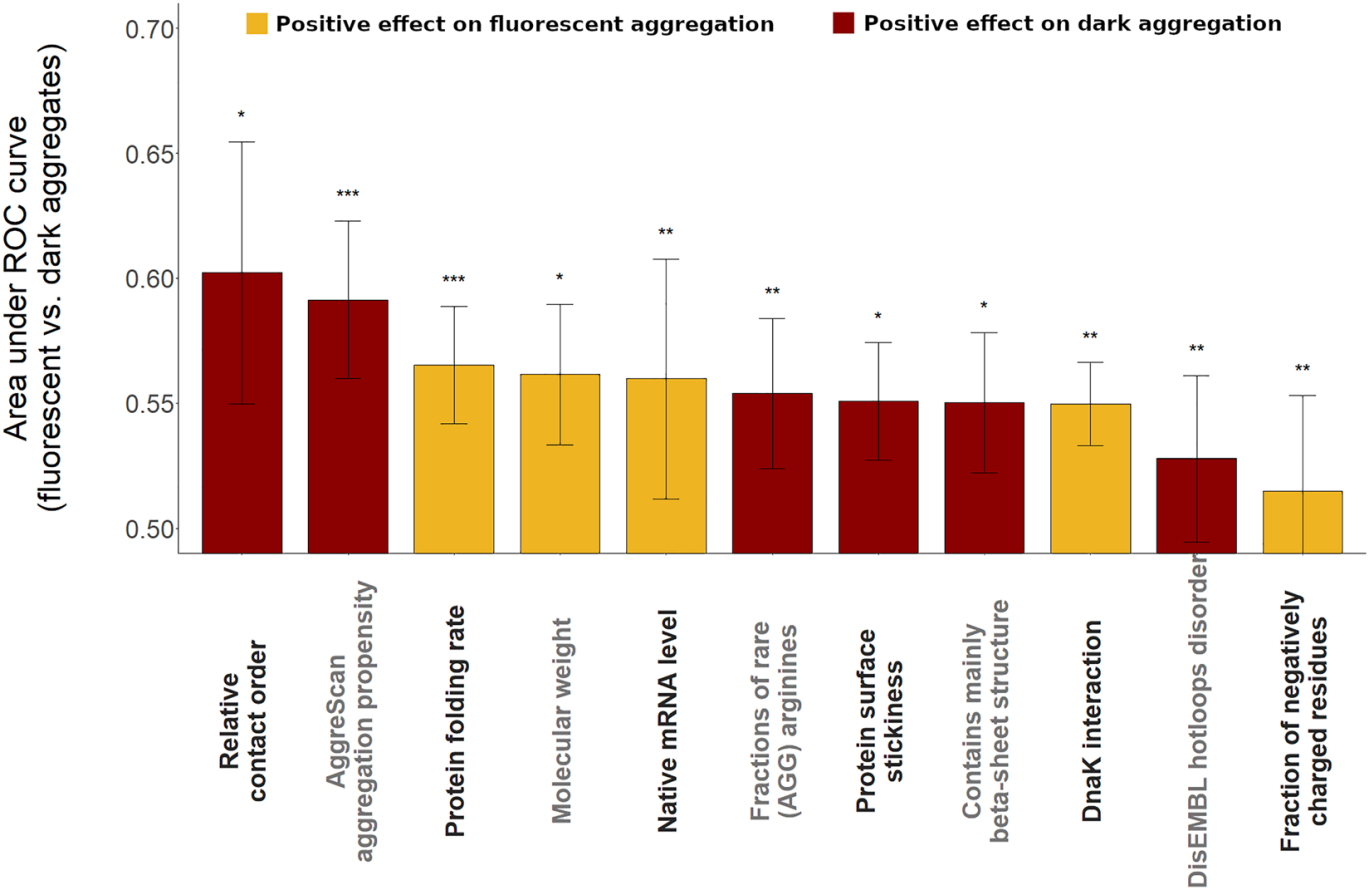
Protein features distinguishing between slowly and rapidly aggregating proteins. Features discriminating between proteins that form dark (rapid) versus fluorescent (slow) aggregates. The predictive ability of each feature was measured as the average area under the receiver operating characteristic (ROC) curve in a tenfold cross-validation procedure based on logistic regression analyses. All displayed protein features are statistically significantly predictive after adjustment for multiple testing using the false discovery rate method, ** corresponds to p_adj < 0.01, * to p_adj < 0.05 (logistic regression). Error bars show the 95% confidence interval for the AUC value of each feature. Note that additional features with weaker discriminating ability are listed in Suppl. Table S5.

We next sought to systematically uncover the molecular features distinguishing between rapidly and slowly aggregating proteins. To this end, we compiled a dataset of 115 protein features describing various physicochemical, structural and functional genomic properties for the vast majority of cytoplasmic *E. coli* proteins (see Table S4). Notably, predicted three-dimensional structures are available for 93% of cytoplasmic proteins in *E. coli*^24^. We focused on monomeric proteins only (N = 1631) to avoid potential biases arising from oligomer (heteromer or homomer) interfaces^25^. Next, we probed each feature for its ability to discriminate proteins that form dark and fluorescent aggregates from each other using logistic regression tests (“Methods”). The analysis revealed major differences between proteins in dark versus fluorescent aggregates (Fig. 3, Table S5).

**Figure 4:**
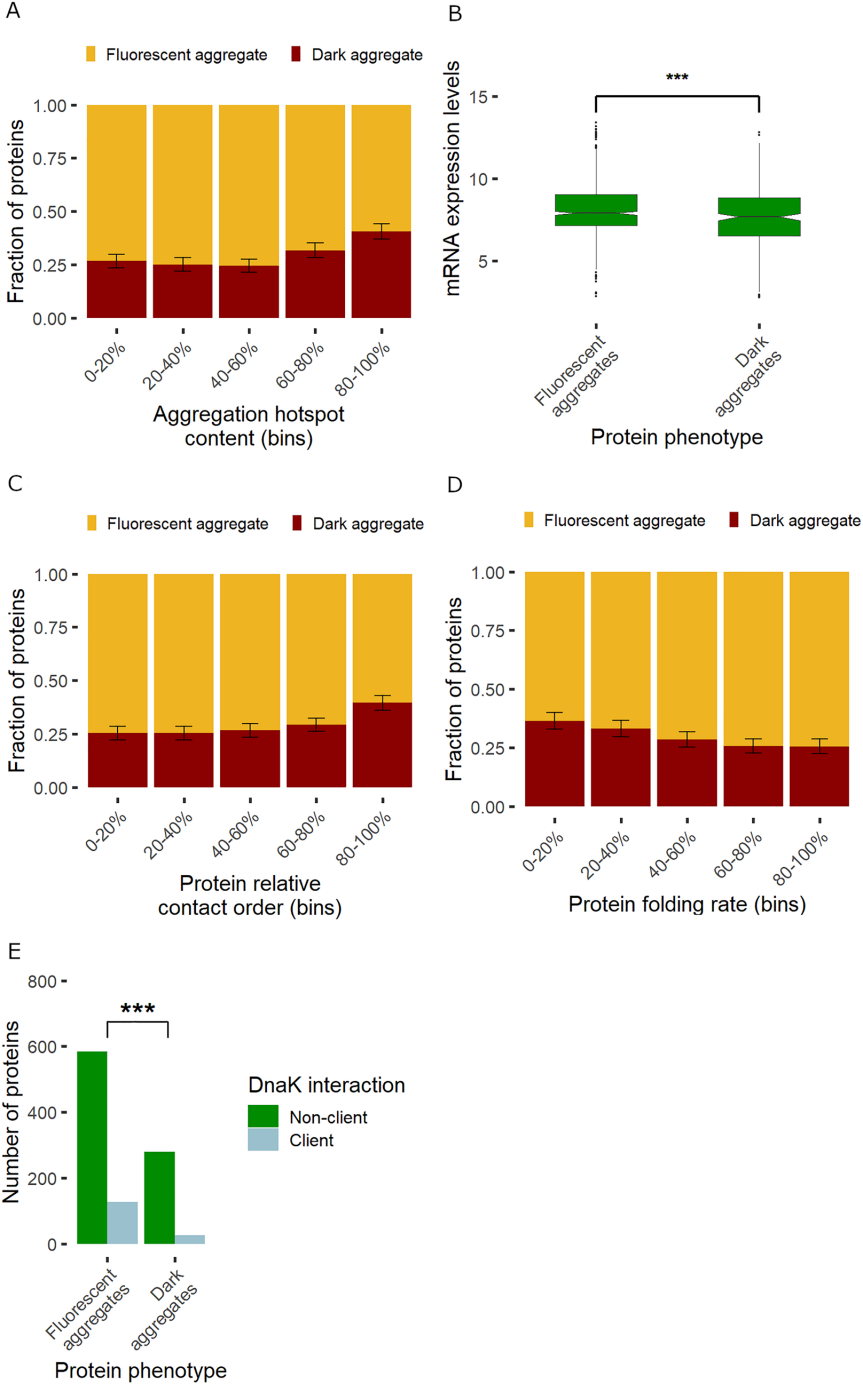
Key molecular features associated with in vivo aggregation rate. (**A**) Proteins with more residues in aggregation hotspots, as estimated by AggreScan^26^, are more likely to form dark (i.e. rapid) than fluorescent (i.e. slow) aggregates (P = 5.01*10^–7^, logistic regression). (**B**) Native mRNA expression levels of proteins in fluorescent aggregates are significantly higher than those in dark aggregate (P = 0.0008, Wilcoxon rank-sum test). (**C**,**D**) Effects of protein contact order and folding rates on the class of aggregation. Note that a lower contact order and a higher folding rate (FOLD-RATE score) indicate easier folding. Dark aggregates are associated with a lower folding ability. (**E**) Proteins in fluorescent aggregates are enriched in DnaK chaperone clients compared to those in dark aggregates (P = 4.94*10–5, Odds ratio = 2.43, Fisher’s exact test). Whiskers show standard errors and were calculated by bootstrap resampling.

As expected, proteins in dark aggregates show several features that have been associated with high aggregation rates. In particular, proteins with more extensive aggregation hotspots (i.e. aggregation-prone sequence regions) are overrepresented among proteins in dark aggregates compared to fluorescent aggregates (Fig. 4A, P = 5.01*10^–7^, logistic regression, based on the AggreScan^26^ predictor, see Table S5 for other predictors). This is in line with previous results showing that the presence of aggregation hotspots increases the rate of aggregation from the unfolded state^19,27^. Proteins with high expression levels generally display low intrinsic aggregation propensities^28^. Consistent with this notion, we found that proteins in fluorescent aggregates show higher native mRNA levels than those in dark aggregates, indicating that they have evolved lower in vivo aggregation rates (Fig. 4B). Overall, these results support the notion that fluorescent and dark aggregates correspond to slow and fast aggregation, respectively.

More remarkably, we found that proteins in fluorescent aggregates tend to have higher folding rates, as indicated by two independent estimates (Figs. 3 and 4C,D). We approximated folding rate by Fold-Rate^29^, an ensemble predictor based on folding-correlated features derived from the primary sequence, and contact order^30^, which measures sequence separation between contacting residues in the native state and is inversely correlated with folding rate (Methods). Both folding rate and contact order distinguishes between proteins in the two classes of aggregates (P = 0.0055 and P = 0.004 by logistic regressions for folding rate and contact order, respectively, Fig. 4C,D). Notably, contact order is the most predictive of the aggregation class out of the 115 tested protein features (Fig. 3). Taken together, these findings indicate that slowly aggregating proteins tend to exhibit high folding rates. Owing to kinetic competition between aggregation and folding events, these results also suggest that some of the proteins displaying fluorescent aggregates may complete initial folding before aggregation takes place^19^.

Finally, we report that proteins in fluorescent aggregates are more likely to be DnaK clients than those in dark aggregates (P = 4.95*10^–5^, Fisher’s exact test, Fig. 4E). DnaK is the major bacterial Hsp70 that interacts with hundreds of cytosolic proteins, most of which are assisted in their initial folding^10^. As DnaK promotes proper folding and prevents aggregation of client proteins^31^, this result may indicate that proteins in fluorescent aggregates are prevented from rapid aggregation in their unfolded state by DnaK. Alternatively, proteins with relatively low aggregation rates might be intrinsically overrepresented among DnaK clients and their folding cannot be completed upon overexpression owing to limited chaperone availability. Further works are needed to distinguish between these scenarios.

### Determinants of high solubility

Our proteome-wide screen revealed a subset of cytosolic proteins that remain soluble even when expressed well above their normal levels. Such highly soluble proteins are unlikely to be present at concentrations close to their solubility limits under physiological conditions. We next sought to identify the molecular properties that distinguish highly soluble proteins from both groups of aggregating proteins (Methods). Consistent with earlier works, soluble proteins show high mRNA expression levels^32^, high content of negatively charged residues^8,33^, low content of hydrophobic amino acids^34,35^ and small size and surface area^8^ (Figure S4). More remarkably, our analysis identifies low surface stickiness and high protein disorder content as major determinants of solubility.

**Figure 5:**
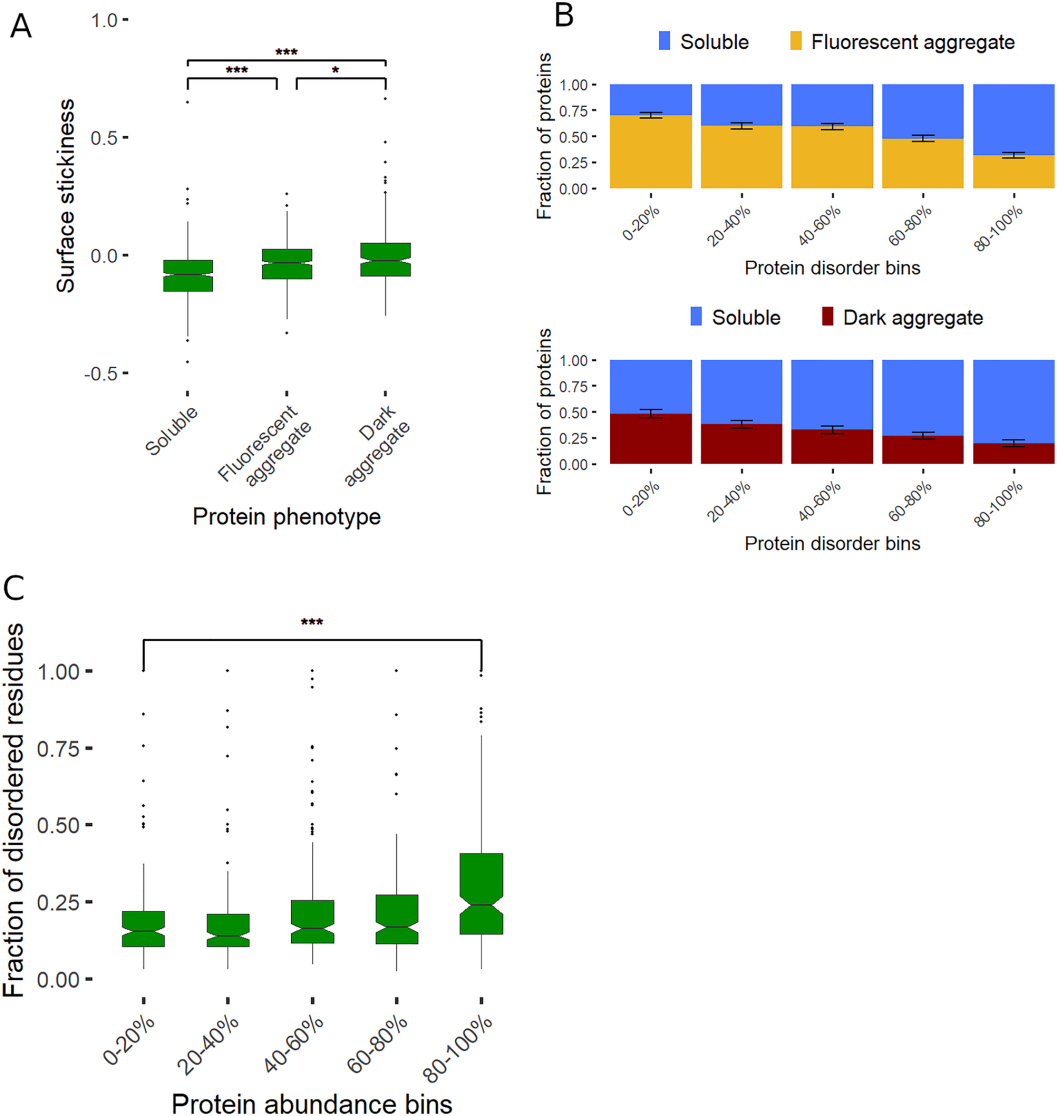
Protein stickiness and disorder content shape solubility. (**A**) We use the surface stickiness score to measure promiscuous interaction propensity^38^. Both fluorescent and dark aggregates show higher surface stickiness than soluble proteins (P < 10^–10^ and P < 10^–10^ respectively, Wilcoxon Rank Sum test). (**B**) Fractions of aggregating proteins as a function of disorder content (binned data), calculated using PONDR VSL2B. Upper panel shows the fraction of proteins in fluorescent aggregates among those that are either soluble or in fluorescent aggregates, while the lower panel shows the fraction of proteins in dark aggregates among those that are either soluble or in dark aggregates. (**C**) Disorder content as a function of native protein abundance in E. coli based on^41^. The most abundant 20% of E. coli proteins have a significantly higher disorder content than those in the least abundant 20% bin (P = 4.18*10^–11^, Wilcoxon rank sum test). Disorder content was calculated using PONDR VSL2B, but similar results are obtained with other predictors (see Tables S6 and S10).

Proteins differ in their propensity to form non-specific interactions with other macromolecules depending on the ‘stickiness’ of their surfaces^36–38^. While low stickiness has been linked to avoidance of non-functional interactions^38^, its role in avoiding aggregation has remained unclear. We find that soluble proteins show a markedly lower surface stickiness score than either class of aggregates (P < 10^–10^ in both cases, Wilcoxon Rank Sum test*,* Fig. 5A, see Methods). Notably, while highly abundant proteins tend to have less sticky surfaces^38^, the association between high solubility and low stickiness remains when controlling for abundance (P = 2.38*10–7 and P = 0.00946 for fluorescent and dark aggregates, respectively, logistic regressions).

Proteins often contain intrinsically disordered regions that lack a unique structure^39^. We find that the proportion of disordered residues (disorder content) of a protein is strongly associated with its solubility upon overexpression (Fig. 5B). To examine the impact of disordered regions on this association, we next identified segments of contiguously disordered amino acids (≥ 10 residues). We find that soluble proteins contain both more such disordered segments per length and a higher proportion of disordered residues outside these segments than those forming either class of aggregates (Table S6). These results are robust to using different predictions of disorder (Tables S6 and S7). Several protein properties that have been associated with intrinsic disorder also influence solubility and therefore may confound the above result. For instance, disordered residues tend to be less hydrophobic and more charged^27,35^, properties that also enhance solubility^33^. However, the association between disorder content and solubility remains when controlling for these properties (Table S8). Furthermore, our analyses show that both disorder content and surface stickiness have independent effects on solubility (Table S9).

If disorder content and protein stickiness shape protein solubility at native expression levels as well then highly abundant proteins should exhibit high disorder content and low surface stickiness as evolutionary adaptations to enhance their solubility. Indeed, it has been reported that highly expressed proteins in *E. coli* exhibit low surface stickiness^38^ and elevated disorder content^40^, albeit these patterns have not been linked to protein solubility. By analyzing the same set of cytosolic proteins for which aggregation phenotype was measured and using state-of-the-art proteomics data on native protein levels^41^, we confirm these correlations (Table S10). Importantly, the correlation between abundance and disorder is not confounded by potential differences in hydrophobicity and charge of residues between high- and low-abundance proteins (Table S10). Furthermore, this association is mainly caused by elevated disorder in the most abundant group of proteins (Fig. 5C), which is consistent with the notion that such proteins are highly optimized to avoid aggregation.

Collectively, these results suggest that elevated structural disorder and low surface stickiness enhance protein solubility in the bacterial cell and play important roles under physiological conditions.

## Discussion

Here we systematically estimated the in vivo solubility and relative aggregation time of *E. coli* proteins by overexpressing them and monitoring their fate using a C-terminally fused GFP reporter. We have identified on a proteome-wide scale those proteins that (i) remain soluble, (ii) aggregate rapidly before the fused GFP is folded, potentially as early as being synthesized, and (iii) those that aggregate more slowly, after being synthesized (i.e. the downstream GFP tag is fully synthesized and folded). Our image-based screen revealed that the majority (70%) of aggregation-prone cytosolic proteins show evidence of aggregation after being synthesized. Note that this is likely to be an underestimate because some of the rapidly aggregating proteins might aggregate post-translationally but before complete folding of the downstream GFP tag. We note that some overexpressed proteins showing no fluorescent signal might not be expressed or degrade due to their limited stability or low half-lives. However, such cases must be rare because over 90% of a representative sample of non-fluorescent proteins were detected by western blot analysis.

We found several key differences in the molecular properties of rapidly and slowly aggregating proteins. First, as might be expected, proteins that show slow aggregation in vivo tend to have lower contents of aggregation hotspots compared to rapidly aggregating ones, indicating that they exhibit lower intrinsic aggregation rates from the unfolded state^19,27^. Such proteins also display higher mRNA expression levels, suggesting that their lower intrinsic aggregation rate arises from evolutionary pressure to avoid aggregation at concentrations required for optimal function^32^. Second, and more strikingly, we found that slowly aggregating proteins exhibit higher predicted folding rates compared to rapidly aggregating ones. Specifically, a key proxy for folding speed is contact order, i.e. the average sequence separation between contacting residues in the native protein structure^30^ and we show that slowly aggregating proteins have lower contact orders than rapidly aggregating ones. As proteins with a low contact order tend to fold co-translationally, these results indicate that proteins that fold rapidly and / or co-translationally are more likely to form aggregates after synthesis^42^. Furthermore, we found that slowly aggregating proteins are also more likely to be clients of DnaK, a chaperone with major role in initial folding of nascent polypeptides^10^. Together, these findings are consistent with the notion that kinetic competition between proper folding and aggregation from the unfolded state influences the conformational state from which aggregation occurs. Prior works showed that a relatively slow aggregation and rapid folding of a protein may permit its folding before aggregation occurs^19^. Therefore, we speculate that at least some of the slowly aggregating proteins might form aggregates from the folded state. As the majority of aggregation-prone cytosolic proteins aggregate after translation in our screen, these reasoning imply that aggregation from the folded state might be an important source of overexpression-induced insolubility. Critically, future studies are needed to test this hypothesis as our GFP-based assay provides no direct information on the folding state of proteins at the time of aggregation. If inclusion body formation frequently occurs from a folded state, it would raise the possibility that some of the inclusion bodies contain catalytically active proteins^4,43,44^. This intriguing possibility could be systematically investigated by combining automated high-throughput inclusion body purification systems^45^ with large-scale functional profiling of enzymes^46,47^.

Our study provides new insights into molecular determinants of high protein solubility in the crowded intracellular environment. First, our results suggest that non-specific protein–protein interactions play an important role in shaping the solubility limits of proteins. It has been shown that natively abundant proteins have evolved an especially low surface stickiness to avoid non-specific interactions and hence interference with other proteins^36,38^. Our work goes further and demonstrates that proteins with low surface stickiness also tend to avoid aggregation. Based on these observations, we propose that avoidance of non-specific interactions is an important mechanism to reduce the aggregation propensity of highly expressed proteins. Second, we show that disorder content is a major driver of solubility differences between *E. coli* proteins in vivo independently of the effect of surface stickiness. Importantly, high disorder content is associated with solubility regardless of the overall charge and hydrophobicity of the proteins (Table S9), suggesting that it is the flexibility conferred by disordered regions what matters for enhanced solubility. This notion is consistent with prior works on the role of flexible structural elements in protein solubilization^48,49^. Specifically, fusion of disordered segments to insoluble proteins have been shown to aid protein folding and solubilization by providing favorable surface area and by acting as “entropic bristles”^49^. Our results also shed new light on how natural selection shapes the disorder content of proteins. It has been noted earlier that highly expressed proteins display elevated disorder content in *E. coli*, however, the underlying selective constraints have remained puzzling^40^. Our results indicate that disorder content has evolved partly to avoid aggregation of highly abundant proteins under physiological conditions in *E. coli*. Thus, we propose that both low surface stickiness and elevated disorder content contribute to the high solubility of abundant proteins in bacteria^32^.

Finally, our screen shows that about one third of cytosolic proteins in *E. coli* remain substantially soluble even when strongly overexpressed. This finding implies that these proteins are unlikely to be supersaturated under physiologically relevant conditions. This conclusion is broadly consistent with a prior proteomic study showing that about one quarter of proteins are expressed below their critical concentrations in *C. elegans* under non-stressed conditions^2^. Importantly, our finding also has relevance to the ‘life on the edge’ hypothesis, which posits that proteins have evolved to be sufficiently soluble to allow their expression at the levels needed for their biological roles, but have almost no margin of safety to tolerate elevated concentrations^1,2^. Contrary to this hypothesis, the critical concentrations of soluble proteins identified in our screen appear to be much higher than those required to maintain their solubility under normal conditions. Thus, avoidance of supersaturation may not be the only evolutionary force affecting protein solubility for a subset of cytoplasmic proteins. We speculate that extremely high solubility might have evolved indirectly as a by-product of selection on other protein features that also influence solubility. For example, low protein surface stickiness might have primarily evolved to minimize dysfunctional, promiscuous protein interactions^38^, but also enhances solubility as a by-product. Clearly, further studies are needed to decipher the evolution of extremely high critical concentrations.

## Materials and methods

### Image-based high-throughput screen of protein aggregation

We used the C-terminal GFP fusion version of the *E. coli* K-12 Open Reading Frame Archive library^22^ to probe the aggregation pathways of 2577 native *E. coli* proteins in an image-based screen. Part of this screen was previously published and included a set of 611 *E. coli* proteins that form homomers and we used a similar methodologyin our image-based analyses^9^. In this previous work, we distinguished only two aggregation phenotypes (homogeneous GFP signal throughout the cells indicating a folded and soluble protein), and proteins without a GFP signal indicating aggregation before reaching the native conformations (that is, a “dark” aggregate). Here, we substantially extended this earlier dataset in two ways. First, here we also included homomers that show fluorescent foci and therefore represent inclusion bodies with properly folded C-terminally fused GFP (that is, a “fluorescent” aggregates). Second, we carried out the screen for the rest of the *E. coli* K-12 proteome. The applied protocol was as follows.

### Cell preparation

Cells were prepared for the image-based screen as described before^9^. In brief, the C-terminal GFP fusion version of the E. coli K-12 Open Reading Frame Archive library^22^ was grown in the original host strain *E. coli* K-12 AG1 in 96-well plates (growth conditions: 37 °C, 280 rpm, LB medium supplemented with 20 µg/ml chloramphenicol as a selection marker of ASKA plasmids). We emphasize that this version of the GFP molecule is optimized to be highly expressed and show high fluorescence intensity in *E. coli* growing at 37 °C^22^. Following overnight growth, expression was induced for 2 h by 0.1 mM IPTG in the fully-grown culture at 37 °C. From the induced cultures 0.2 μL were carried over using a pin tool replicator into black CellCarrier-96 plates (PerkinElmer). In this plate, each well had been supplemented with 100 μl of 5 μg/mL 4,6-diamidino-2-phenylindole (DAPI) in mineral salts minimal medium (MS-minimal) without any carbon source. Prior the microscopic analysis, cells were centrifuged down to the bottom of the 96 well plates.

### Imaging

Microscopy was done using a PerkinElmer Operetta microscope as established previously^9^. Four sites were acquired per well. Laser-based autofocus was performed at each imaging position. Images of two channels (DAPI and GFP) were collected using a 60 × high-NA objective to visualize the cell and the aggregation states of the proteins, respectively. At every site and every fluorescent channel 5 images were taken at different z positions with 0.5 μm shifts. These images were used for a perfect focus algorithm. Cellular properties of about 1000 cells of each expressing strain were extracted from the images, including the localization of the GFP signal within the cell.

### Image analysis

Image analysis was conducted according to previously described methodology^9^. Images were pre-processed using the CIDRE algorithm^50^ to remove uneven illumination. A perfect focus algorithm was developed to locally select the best z image plane and create an image that contains the highest contrast cells. To identify cells and extract their properties, the CellProfiler program^51^ was used with custom modifications. First, image intensities were rescaled. Then, cells were identified on the DAPI signal using Otsu adaptive threshold and a Watershed algorithm to split touching cells. Cellular features such as intensity, texture, and morphology were extracted. The raw microscopy data can be accessed at http://group.szbk.u-szeged.hu/sysbiol/scientific-resources/Proteome-wide-landscape-of-solubility-limits-in-a-bacterial-cell/Proteome_wide_landscape_of_solubility_limits_in_a_bacterial.zip.

### Phenotypic classification using machine learning

Supervised classification of cells into predefined groups was performed using the Advanced Cell Classifier software^52^. The cellular phenotypes were (i) no GFP signal (fluorescence level equals to that of the negative control without GFP) (ii) homogenous GFP signal (cells show equally distributed GFP signal throughout the whole cell) (iii) concentrated GFP signal in either one or both poles of the cell. Cells that did not fit into these three categories were discarded. For the automated decision, an artificial neural network method was used based on the Weka software^53^.

Based on this cell classification, the proteins were assigned to one of the three classes, depending on which phenotype was predominant in the cell population. We considered a protein as “Dark” if the most populous category of cells showed no fluorescence. If the predominant cellular phenotype was a concentrated fluorescent spot at the cell pole, proteins were classified as “Fluorescent foci”, and if the majority of cells showed diffuse green fluorescence, proteins were classified as “Diffuse fluorescent”.

### Protein expression analysis

We carried out western blot analyses for a representative set of protein overexpressions from the three groups of aggregation phenotypes with a special focus on the expression levels of the ‘dark aggregate’ group (i.e. those not showing a fluorescence signal). In brief, the C-terminal GFP fusion version of the *E. coli* K-12 Open Reading Frame Archive library (ASKA), members were grown overnight in 1 ml LB medium supplemented with 20 µM chloramphenicol at 37 °C^22^. Following overnight growth, expression was induced for 2 h by 0.1 mM IPTG in the fully-grown culture at 37 °C. Following expression, cells were harvested by centrifugation (~ 13,000 g) and the pellets were resuspended in 250 µl 2×SDS-sample buffer. After boiling the samples for 5 min, 5 μl were separated on 10% SDS–polyacrylamide gel (PAGE). Gels were either stained with Coomassie Brilliant Blue (CBB) for justifying equal loading or transferred onto PVDF membranes (Amersham, GE Healthcare Lifescience) proceeding further for western blotting. Next, membranes were blocked in 5% (w/v) milk powder-0.05% (v/v) Tween20 in TBS (25 mM Tris–Cl, pH 8.0, 150 mM NaCl) buffer (TBST) for an hour at room temperature (RT). Next, the membranes were incubated with 5% (w/v) milk powder-TBST including anti-GFP (Chromotek) as primary antibody (diluted to1:1000) and agitated overnight at 4 °C. After washing three times with TBST buffer to remove the excess of unbound primary antibody, membranes were incubated with appropriate secondary antibody (Sigma-Aldrich) diluted in 2.5% (w/v) milk-powder-TBST buffer (1:10000) for an hour on RT. After washing the membranes three times in TBST buffer, signals were developed by a standard chemiluminescent western blot detection method (Thermo Scientific).

### In vivo solubility analysis

Next, we tested the aggregation propensity of a representative set of protein over-expressions to confirm the predicted aggregation phenotypes coming from the microscopic image analyses. In brief, cells were grown as described above. After the centrifugation step, cell pellets were lysed by resuspending them in 200 μl BugBuster (Sigma) reagent at room temperature. Cell suspensions were incubated on a shaking platform at a slow setting for 20 min at room temperature. The solutions were centrifuged at 16,000×*g* for 20 min at 4 °C. 200 μl soluble fractions were removed and 50 μl 5×SDS-sample buffer was added. The pellets were resuspended in 250 µl 1 × SDS-sample buffer. After boiling the samples for 5 min, 5 μl were separated on 10% SDS–polyacrylamide gel (PAGE). Western blot was carried out as described above. Signals were converted into black and white images and then quantification of the western blot bands (degradation products were not counted) was carried out by Image Studio Light. Band area was then corrected by eliminating the background value.

### Bioinformatics analyses

Each protein was assigned a cellular localization according to StepDB^54^, oligomerization data was retrieved from EcoCyc^55^. Only cytoplasmic monomers were used in further calculations, resulting in 1631 proteins. We collected 115 features for each protein, including mRNA level and protein abundance, presence of protein–protein interactions, disorder content, physico-chemical and functional properties from various sources (see Table S5 for a complete list of protein features and their literature sources).

Predicted protein structures were retrieved from the Zhang Lab webpage (https://zlab.bio/)^24^ with an in-house Perl script, using the package UserAgent version 6.07. The vast majority of protein structures were template-based predictions, only those with no substantial homology to known structures were the product of in ab initio structural predictions. The structures were subsequently used in determining solvent accessible amino acids of the protein using AREAIMOL from the CCP4 suite^56^. Amino acids were classified as solvent accessible, if the sidechain had a solvent accessibility greater than 25%, the default cutoff. Protein stickiness was calculated as the average amino acid stickiness of surface amino acids. We also employed the same structures in calculating contact order^30^, however, contact order can only reliably be calculated for single domain monomers. Similarly, only single domain monomeric proteins were used for folding calculations with FOLD-RATE^29^ with default parameters, yielding 661 proteins in both cases.

Protein function was retrieved from the COG and MultiFun databases. Enzyme Commission numbers were retrieved from the UniProt database. Protein structure classification was retrieved from the CATH database^57^ and secondary structure superfamilies and families were used. Protein disorder data was retrieved from the MobiDB database^58^ and calculated with several different predictors: DisEMBL^59^, PONDR VSL2B^60^, IUpred^61^ and Espritz^62^ with default parameters to account for different biases that might be present in any single predictor. The number of disordered amino acids was normalized to the protein length in all cases. Disordered protein segments were defined as segments with continuous disordered residues of length >  = 10 as inferred by each individual predictor. Basic protein sequence features and amino acid composition was counted and calculated using in-house Perl scripts from the protein sequences.

We used logistic regressions to test the statistical association between aggregation class and each protein feature individually. To measure the predictive power of each protein feature in a comparable manner, we calculated the area under receiver operating characteristic curve for each logistic regression model using the pROC R package, with tenfold cross validation. Furthermore, we used multivariate logistic regression modelling to test the effects of multiple protein features simultaneously and statistically control for each other’s effect.

All downstream calculations were performed in R version 3.5.0, 2018-04-23^63^ in Rstudio version 1.1.447, figures were created in R base and ggplot2 version 2.2.1.

## Supplementary Information

Supplementary Information 1.

Supplementary Information 2.

Supplementary Information 3.

Supplementary Information 4.

Supplementary Information 5.

Supplementary Information 6.

Supplementary Information 7.

Supplementary Information 8.

Supplementary Information 9.

Supplementary Information 10.

Supplementary Information 11.
