# Supplementary material for: Proteome-wide landscape of solubility limits in a bacterial cell
Source: Sci Rep. 2022 Apr 21;12:6547. doi: 10.1038/s41598-022-10427-1 (PMC9023497; doi:10.1038/s41598-022-10427-1)
Supplement: Supplementary file 1 — Supplementary Information 1. [file 41598_2022_10427_MOESM1_ESM.docx]

**Supplementary Figures**


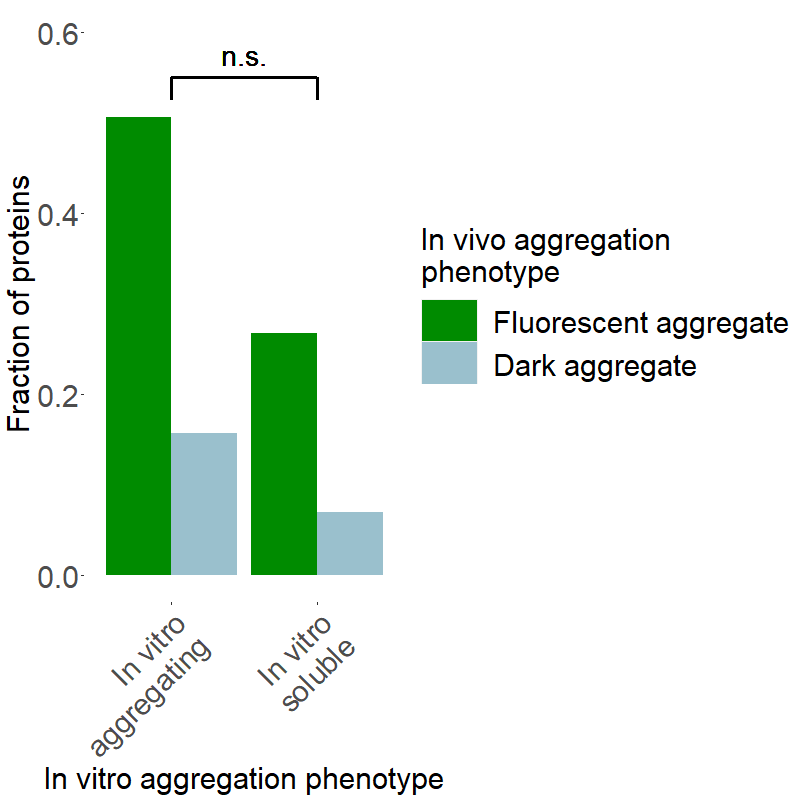


***Figure S1.*** ***The two in vivo aggregation phenotypes show similar overlaps with in vitro aggregation.*** *The relative frequencies of fluorescent versus dark aggregates are similar in the in vitro aggregating and in vitro soluble classes of proteins (P = 0.3287, Fisher’s exact test).*


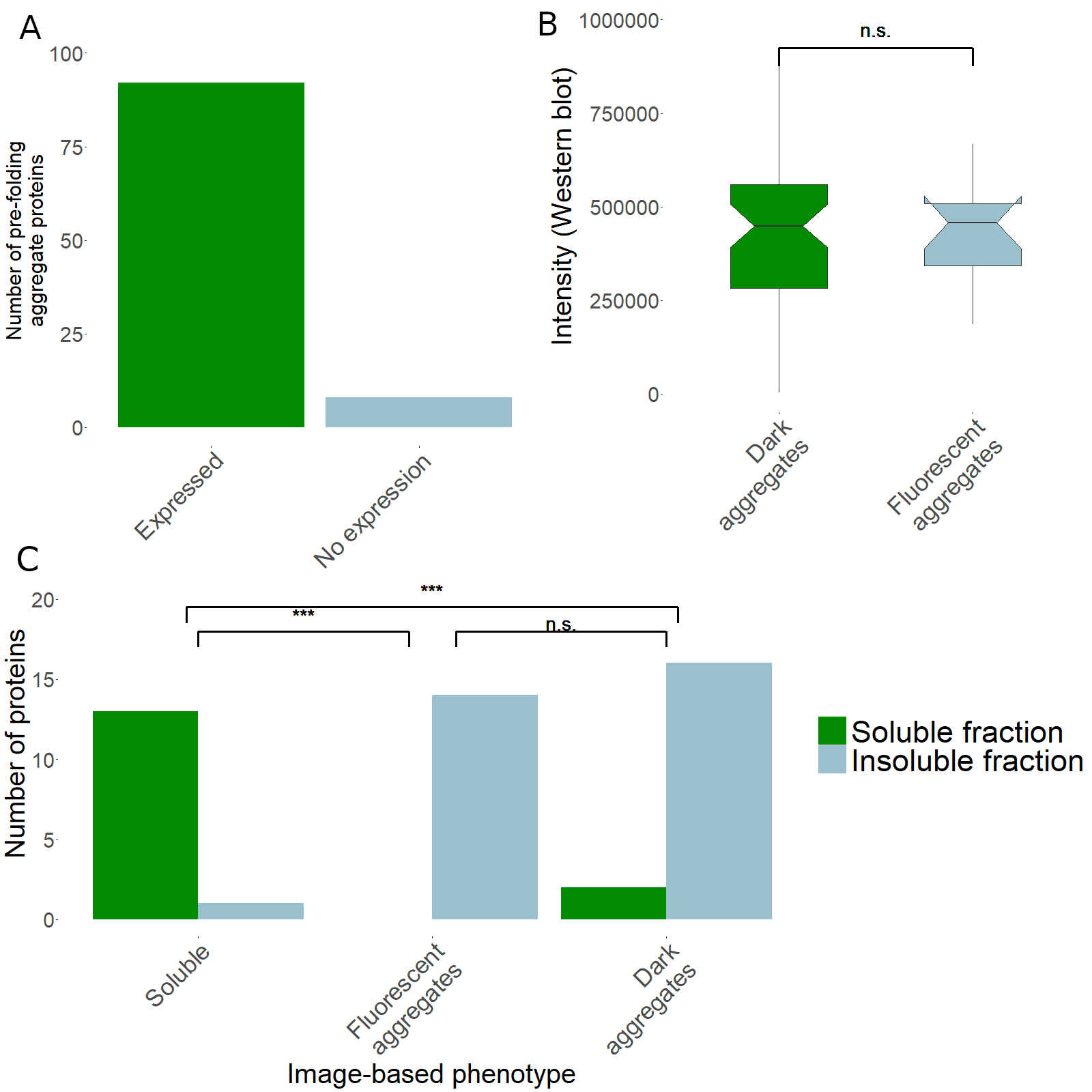


***Figure S2. Experimental validation of protein overexpression and solubility phenotypes. (A)*** *Summary of western blot analysis of a representative subset of 100 proteins classified as ‘dark aggregate’ in the image-based in vivo screen. 92% of these proteins are expressed as detected using western blot.* ***(B)*** *Quantified band intensity measurements from western-blot analysis of fluorescent and dark aggregate proteins. No significant difference was found between the band intensities (P = 0.8794, Wilcoxon Signed rank test, N = 57 and N = 14 for dark aggregate and fluorescent aggregates respectively, see supplementary methods).* ***(C)*** *Validation of the high-throughput image-based cell classification by analyzing the soluble and insoluble protein fractions of a representative set of overexpression strains with SDS-PAGE and western blotting. The set of overexpression constructs represent 14, 14 and 18 proteins that show soluble, fluorescent aggregate and dark aggregate phenotypes, respectively, in the image-based screen. Proteins were classified as aggregate if no protein was detected in the soluble fraction using SDS-PAGE western blot (see Methods). Proteins showing solubility in the image-based analysis are significantly enriched in the soluble fraction compared to proteins that form fluorescent and dark aggregates (P = 7.48*10^-7^ and P = 5.26*10^-6^, respectively, Fisher’s exact test, N = 46). Proteins in fluorescent and dark aggregates show similar relative frequencies of protein aggregation (P = 0.49, Fisher’s exact test).*


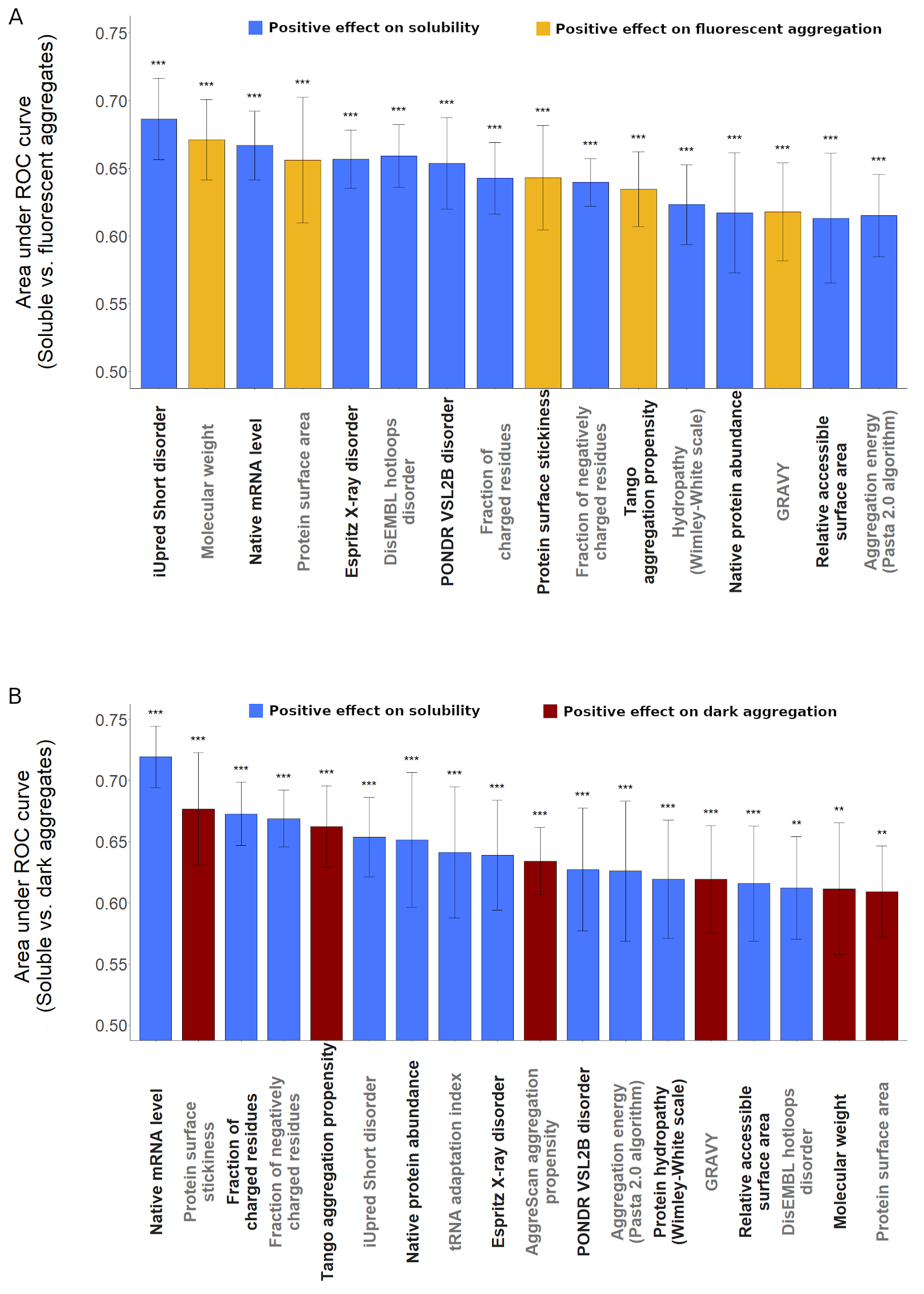


***Figure S3. Major protein features discriminating soluble proteins from the two classes of aggregates.*** *Features discriminating between proteins that remain soluble and those that form fluorescent (A) or dark (B) aggregates, respectively. The predictive ability of each feature was measured as the average area under the receiver operating characteristic (ROC) curve in a tenfold cross-validation procedure based on logistic regression analyses. All displayed protein features are statistically significantly predictive after adjustment for multiple testing using the false discovery rate method,*** corresponds to p adj <0.0001, ** corresponds to p adj < 0.01, * to p adj < 0.05 (logistic regression). Error bars show the 95% confidence interval for the AUC value of each feature.*

Supplementary table legends

Table S1.

List of *E. coli* proteins used in the high-content microscopy experiments and the number (and fraction) of cells showing the distinct phenotypes. Column C shows the final phenotype assigned to each protein based on a majority rule of the cellular phenotypes. Raw microscopy data can be downloaded from <http://group.szbk.u-szeged.hu/sysbiol/scientific-resources/Proteome-wide-landscape-of-solubility-limits-in-a-bacterial-cell/Proteome_wide_landscape_of_solubility_limits_in_a_bacterial.zip>.

Table S2.

List of proteins used in western blot experiments to verify proper protein expression and product size of a randomly selected subset of the ASKA library clones.

Table S3.

Results of the experimental verification of the image-based protein solubility phenotype of a randomly selected subset of proteins.

Table S4.

(A) List of protein properties used in downstream calculations and references to their origins.

(B) Values of the protein properties used in downstream calculations.

Table S5.

Results of the logistic regression models aimed at identifying parameters to differentiate between proteins of different solubility and aggregation phenotypes. (A) Comparison of soluble and fluorescent aggregate, (B) Fluorescent aggregate and dark aggregate and (C) soluble and dark aggregate proteins.

Table S6.

Logistic regression models showing differences in the presence of short and long disordered segments in proteins with the different solubility and aggregation phenotypes. (A) Comparison of soluble and fluorescent aggregate, (B) Fluorescent aggregate and dark aggregate and (C) soluble and dark aggregate proteins.

Table S7.

Logistic regression models showing enrichment of protein disorder in soluble proteins compared to (A) fluorescent aggregate and (B) dark aggregate proteins.

Table S8.

Logistic regression models showing enrichment of protein disorder in soluble proteins compared to (A) fluorescent aggregate and (B) dark aggregate proteins independently of different physico-chemical parameters to control for their effect.

Table S9.

Logistic regression models showing the independent effect of higher protein disorder and lower protein stickiness on solubility for soluble proteins compared to (A) fluorescent aggregate and (B) dark aggregate proteins based on different protein disorder metrics.

Table S10.

Partial correlations between different metrics of protein disorder and protein abundance while controlling for physico-chemical parameters, namely (A) charged residues and (B) hydrophobicity.
